# Coeliac screening in a Scottish cohort of children with type 1 diabetes mellitus: is DQ typing the way forward?

**DOI:** 10.1136/archdischild-2015-309754

**Published:** 2015-12-30

**Authors:** R T Mitchell, A Sun, A Mayo, M Forgan, A Comrie, P M Gillett

**Affiliations:** 1 MRC Centre for Reproductive Health, The Queen's Medical Research Institute, The University of Edinburgh, Edinburgh, UK; 2 Departments of Paediatric Diabetes (RTM) and Paediatric Gastroenterology (PMG), Royal Hospital for Sick Children, Edinburgh, UK; 3 Departments of Paediatric Diabetes, Royal Aberdeen Children's Hospital, Aberdeen, UK; 4 BTS Tissue Typing, Ninewells Hospital, Dundee, UK

**Keywords:** Coeliac Disease, Human Leucocyte Antigen, Screening, Type 1 Diabetes Mellitus

## Abstract

**Background:**

Children with type 1 diabetes mellitus (T1DM) are at increased risk of coeliac disease (CD). Recent guidelines indicate coeliac screening should include HLA typing for CD predisposing (DQ2/DQ8) alleles and those negative for these alleles require no further coeliac screening.

**Methods:**

Children (n=176) with T1DM attending clinics across two Scottish regions were screened for HLA DQ2/DQ8 as part of routine screening. Data collected included the frequency of DQ2/DQ8 genotypes and the additional cost of HLA screening.

**Results:**

Overall, DQ2/DQ8 alleles were identified in 94% of patients. The additional cost of HLA typing was £3699.52 (£21.02 per patient). All patients with known CD (11/176) were positive for DQ2/DQ8 and all were diagnosed with CD within 5 years of T1DM diagnosis.

**Conclusions:**

The vast majority of children with T1DM have CD-predisposing HLA genotypes limiting the number of patients that can be excluded from further screening. We conclude that HLA genotyping is not currently indicated for CD screening in this population.

**What is already known on this topic**

- Coeliac disease is relatively common in children with type 1 diabetes compared with the general population.
- HLA genotyping may be useful in determining the risk of developing coeliac disease.
- Screening for coeliac disease, including HLA genotyping, is recommended for children with type 1 diabetes.

**What this study adds**

- We demonstrate that coeliac predisposing genotypes are present in the vast majority of patients with type 1 diabetes in a UK cohort.
- Screening for HLA genotypes is not currently cost-effective for coeliac screening in patients with type 1 diabetes.
- Clarification of coeliac disease risk for specific HLA genotypes is urgently required for implementing a screening strategy in patients with type 1 diabetes.

## Introduction

Children with type 1 diabetes mellitus (T1DM) are at increased risk of coeliac disease (CD) compared with the general population.^1^ According to current guidance, serological screening for CD is recommended at T1DM diagnosis for adults and children and at ‘regular intervals’ thereafter, although frequency is not specified.^2^ Current practice involves measuring levels of coeliac-related antibodies, most commonly anti-tissue transglutaminase (TTG) and antiendomysial antibodies. Some centres test yearly, others every 2 years or less. There is no robust evidence to guide clinicians or families about the frequency of serological testing for CD.^3^ ^4^ ESPGHAN and BSPGHAN have published guidelines for the assessment of populations at increased risk of CD, including T1DM.^5^ ^6^ They suggest that for patients with associated conditions (including T1DM) the first-line screening should be HLA-DQ typing in addition to anti-TTG. The algorithm indicates that those patients with a negative DQ result will not require any further coeliac screening.^5^ ^6^

The HLA genes are located on chromosome 6 and encode a group of cell surface antigen-presenting proteins. The majority of patients with CD (>90%) carry a variant of HLA-DQ2 (DQ2.5_CIS_). Others carry HLA-DQ8 or HLA-DQ2.2 genotype. The HLA-DQ2.5 antigen is encoded by alleles DQA1*0501 and DQB1*0201 and HLA DQ8 is encoded by alleles DQA1*0301 and DQB1*0302.^7^ ^8^ Around 30% of the general population will have one of the coeliac-associated haplotypes but only 1–2% of the whole population would have CD if screened.^5^ Importantly, <1% of patients with CD lack the predisposing HLA alleles. Superficially, this seems very straightforward; however, conflicting opinion exists, as the genetics are complex.

The HLA-DQ2/DQ8 genotype has recently been reported in up to 86% of Dutch patients with T1DM.^9^ Because these haplotypes occur with reported high frequency in T1DM, it is unclear whether the ESPGHAN/BSPGHAN approach involving HLA-DQ2/DQ8 typing offers real benefit to patients or economic benefit to healthcare providers.

We hypothesised that the proportion of patients with T1DM in our population with a negative haplotype would be low and would limit the patient benefit and cost-effectiveness of the proposed screening strategy in the T1DM population.

## Methods

### Ethics

The study was approved by the local clinical governance team. The South East Scotland Research Ethics Committee was consulted and NHS ethical review was deemed not necessary. Patient data were anonymised prior to analysis.

### Study design

We performed a prospective analysis of the results of coeliac screening in all children aged 1–16 years with T1DM attending the paediatric diabetes clinic in two Scottish regions; Lothian (Royal Hospital for Sick Children, Edinburgh, St John's Hospital, Livingston; n=103) and Grampian (Royal Aberdeen Children’s Hospital, Dr Grays Hospital, Elgin; n=73). Data were collected between January 2014 and January 2015 on consecutive patients. Testing was part of routine practice following initial T1DM diagnosis or as part of annual review. Samples were analysed for HLA-DQ2.5, HLA-DQ2.2 and HLA-DQ8. The frequency of alleles was calculated and the results compared with those of anti-TTG antibodies, biopsy results if they had been biopsied and found not to be coeliac, and those with a confirmed diagnosis of CD.

### Blood sampling

A 2–5 mL EDTA venous blood sample was obtained and sent to the National Screening Laboratory (BTS Tissue Typing, Ninewells Hospital, Dundee, Scotland, UK). Samples were tested for HLA-DQ2.5, HLA-DQ2.2 and HLA-DQ8 using an Immucor Gamma Life codes HLA DQB1* and DQA1*kit and read using a Luminex instrument, which identified HLA DRB1* to two digits (low resolution) and HLA DQA1* to high resolution (four digits). Results were reported as negative (for all alleles) or alleles present were described and interpreted within an executive summary. Anti-TTG was measured at diagnosis or at review and every 2 years as part of routine clinical practice.

### Cost–benefit analysis

The total laboratory cost (including reagents, equipment and staff) of performing the new and existing strategies was calculated. This included the cost of IgA (£3.66) and anti-tTG IgA (£15.92) at diagnosis followed by repeat anti-tTG IgA (£15.92) every 2 years until transfer to adult services at 18 years of age. The additional cost of initial HLA screening (£25) compared with current practice was calculated.

## Results

**Figure 1:**
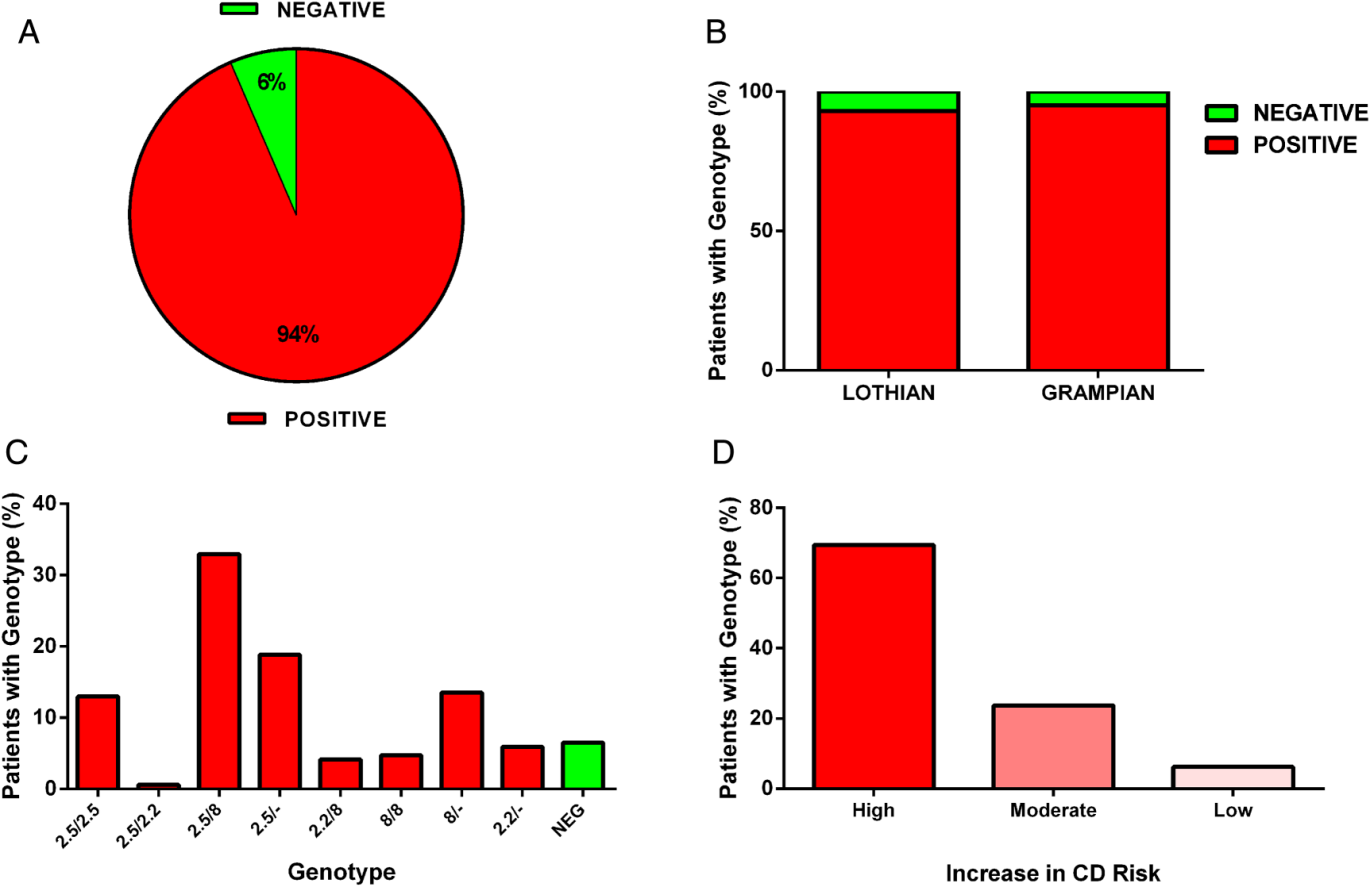
(A) Frequency of coeliac disease (CD)–predisposing HLA (DQ2 and/or DQ8) genotypes in children with type 1 diabetes mellitus. (B) Frequency of HLADQ2 and/or DQ8 by region. (C) Frequency of individual CD predisposing (red bars) and non-predisposing (green bars) haplotypes in the whole cohort. (D) Degree of increased risk of CD based on data from Tye-Din *et al*^10^ for patients positive for a CD-predisposing haplotype. NEG, negative for DQ2 and/or DQ8

Over a 12-month period we screened a total of 176 (56% female and 44% male) children with T1DM for HLA-DQ2.5, HLA-DQ2.2 and HLA-DQ8 status. The median age at screening was 11.56 years. The overall frequency of the predisposing HLA-DQ2 and/or HLA-DQ8 (risk) alleles was 94% (figure 1A). This frequency did not vary between Lothian (93%) and Grampian (95%; figure 1B). The distribution of genotypes is shown in figure 1C with the most frequent genotype, DQ2.5/DQ8, present in 32% of patients. Of those patients with a CD predisposing HLA genotype, 69% carried at least one allele of the ‘high increased risk’ DQ2.5, while 24% carried a ‘moderate increased risk’ (DQ8±DQ2.2) and the remaining 6% carried a ‘low increased risk’ (DQ2.2 only) genotype (figure 1D^10^).

**Figure 2:**
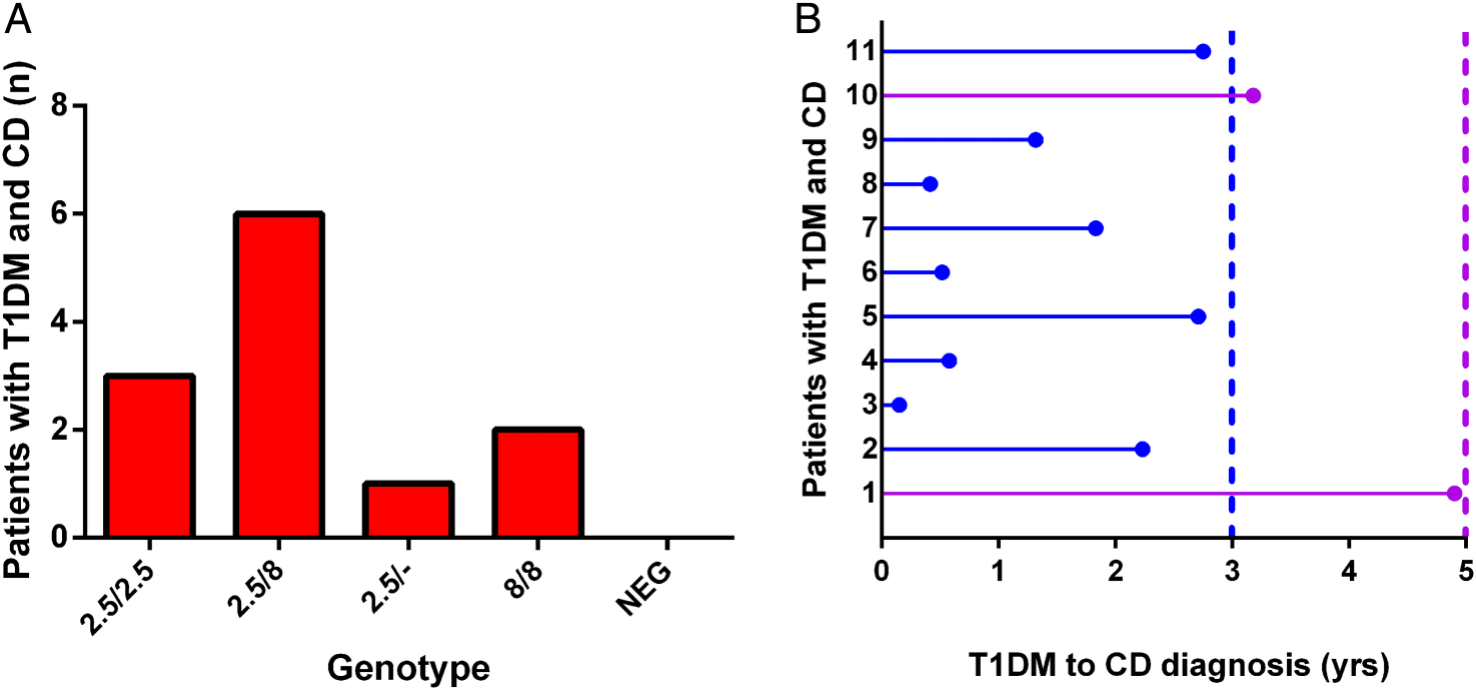
(A) Frequency of DQ2/DQ8 haplotypes in patients with type 1 diabetes mellitus (T1DM) with known coeliac disease (CD). (B) Timing of diagnosis of CD in relation to duration of T1DM. NEG, negative for DQ2 and/or DQ8.

CD was diagnosed in 11/176 (6.9%) patients. Haplotypes reported were DQ2.5/DQ8 (6/11; 55%), DQ2.5/DQ2.5 (3/11; 27%), DQ2.5/- (2/11; 18%) and DQ8/DQ8 (1/11; 9%). Importantly, all 11 patients with CD had a coeliac-related genotype (figure 2A). HLA DQ2/DQ8 testing was highly sensitive (100%) with a negative predictive value of 100% for a diagnosis of CD; however, the specificity (6.7%) and positive predictive value (6.7%) were very low.

We also investigated the time interval from diagnosis of T1DM to diagnosis of CD. The median time from the diagnosis of type 1 diabetes was 2.03 years. All 11 patients were diagnosed with CD within 5 years of their T1DM diagnosis, with 9/11 (82%) diagnosed within 3 years (figure 2B).

We calculated the additional cost of HLA screening in our study population (n=176). The total cost of anti-TTG screening every 2 years (between the ages of 8 and 14 years) is £14 653.76 (£83.26 per patient). Based on a negative HLA-typing frequency of 6%, the cost of anti-TTG screening would be reduced to £13 953.28 (£79.28 per patient). However, the cost of HLA typing for these patients is £4400 (£25 per patient), resulting in a total cost of £18 353.28. This represents an additional cost of £3699.52 (or £21.02 per patient) for this population.

## Discussion

The guidelines produced by ESPGHAN, modified by BSPGHAN,^5^ ^6^ provide a didactic approach that may be used by paediatricians in specialties other than gastroenterology to determine the risk of CD in ‘high-risk’ populations. The guidelines include screening for CD predisposing (DQ2/DQ8) HLA genotypes. In the general population, DQ2/DQ8 is positive in 30–50% of individuals, resulting in a poor positive predictive value for CD; however, 99.6% of those with CD are DQ2/DQ8 positive giving the test a high negative predictive value.^10^ This suggests that the test could be used in ‘high risk’ populations to rule out CD in those who are DQ2/DQ8 negative. Although our results are in keeping with the high negative predictive value described for the general population, we clearly show that DQ typing is positive for DQ2/DQ8 in the vast majority (94%) of paediatric patients with T1DM and therefore in reality this only resulted in 6% of Scottish patients with T1DM being reassured that they do not have the permissibility genes and do not require further testing. Perhaps unsurprisingly, the typing profiles did not vary significantly between the Lothian and Grampian centres, given the stable mix of ethnicity within the Scottish population. The present findings, of a high frequency of DQ2/DQ8 in our T1DM population, are consistent with a Dutch study of 110 children with T1DM, screened with DQ typing in whom DQ2.5 or DQ8 was demonstrated in 86% of patients.^9^ Unlike our study, they did not detail the presence of the DQ2.2 haplotype. The higher frequency in our study was likely due to including the DQ2.2 haplotype as a positive result. We included the presence of a DQ2.2 haplotype as a risk factor (ie, a positive result) although there is some debate about its significance when present alone. There is no doubt that experts agree that DQ2.2 in combination especially with DQ2.5 allows much stronger antigen presentation and greater risk of CD. Two reviews detail DQ2.2 alone as being no risk for CD and a risk only with another haplotype such as DQ2.5.^11^ ^12^ However, a recent paediatric study from Holland confirmed 9 cases of a total 139 (5.8%) who only possessed DQ2.2.^13^

Others suggest non-DQ2/DQ8 haplotypes confer some risk. A study of a total of 1008 patients with CD in a European consortium described 61 (6%) who had neither DQ2 nor DQ8 but who were definitely coeliac and 57 of those 61 confirmed patients with CD had only one half of the DQ2 heterodimer present (DQA1*05 or DQB1*02 but not both). Clearly, patients with non-classical DQ2/DQ8 coeliac genotype must exist, posing a real dilemma when screening and reporting such haplotypes as part of a management strategy. It is clear that there is a real risk of missing the diagnosis by excluding patients from future testing on the basis of DQ2 and DQ8 negativity.^14^ Furthermore, in proven patients with CD from an Australian study, of a total 356 patients, only 1 did not possess either DQ2.5, DQ8 or DQ2.2. Specifically within that cohort, 7 (2%) possessed DQ2.2 only and were definitely coeliac.^15^ Interpreted in another way, the authors concluded in their cohort that at least 99.7% of patients possessed either DQ2.5, DQ8 or DQ2.2. To further evidence this, Harmon *et al*^16^ described 4 of 95 patients with CD who responded to gluten-free diet who carried only DQ2.2. Louka and Sollid^17^ in 2003 very eloquently reviewed the current state of this complex genetic story, the concept of non–HLA gene involvement (undoubtedly important) and the gene dosage effect in relation to relative risk of the condition. Sollid's group detail the difference in peptide recognition between DQ2.5, DQ2.2 and also DQ7.5 and see all as risk factors for the condition, but the different binding may determine the relative risk for each haplotype.^18^

The literature therefore suggests that DQ2.2 constitutes some risk but not no-risk and we would suggest that until further more definitive evidence exists that patients with DQ2.2 are counselled as such. We propose that absolute clarity is required from genetic experts in the field if we are to move forward with using routine DQ typing, in what constitutes ‘risk’ and what constitutes a ‘safe no-risk’ result, given that interpretation as a ‘negative’ test will take that patient out of the screening process. If incorrectly interpreted, this will lead to false reassurance and confusion with families and colleagues.

Given the high frequency of coeliac predisposing haplotypes in children with T1DM, it is important to consider the additional costs of HLA screening. This largely depends on the frequency of serology testing and the cost of HLA typing, which can range from £25 (present study) to £200 (other UK centres; personal communication). The Dutch study assessed cost-effectiveness of including HLA typing, at €166.88 per patient and concluded that the strategy (genotype, total IgA and anti-TTG) was not cost-effective.^9^ The variation in costs of screening in different regions or countries must be taken into consideration when deciding on the most appropriate screening strategy for that population. It is likely that the costs of HLA typing will reduce over time making it a more cost-effective option. In addition, further stratification of CD risk according to specific haplotypes may also improve the effectiveness of CD screening in patients with T1DM. Indeed, risk profiling for CD has been described, with combinations of one or two copies of each allele conferring different relative risks of developing CD.^11^ Future genotyping will be important for those with CD as Nexvax 2 peptide therapy is being developed only for those who are haplotype DQ2.5.^19^

We also demonstrate that after T1DM diagnosis, the majority of patients will have their CD diagnosed within 4 years. Previous work from our group showed that the majority of patients with T1DM with CD have been diagnosed within 5 years of T1DM diagnosis with robust capture of patients during the screening period.^4^ Barera *et al*^3^ report that the majority of presentations of CD in patients with T1DM is within 6 years of T1DM diagnosis, although they documented an increasing reluctance to be tested over time (<10% were tested towards the end of that study). This information may help determine the optimal frequency or duration of screening in T1DM. We suggest that the new ESPGHAN/BSPGHAN strategy, currently being ‘road-tested’ by the Procede group,^20^ may indicate that a rethink needs to take place because in practice it may not prove cost-effective and may raise unrealistic expectations within certain ‘at-risk’ groups.

## Conclusion

DQ typing is readily available within the UK and is recommended by ESPGHAN/BSPGHAN as part of the initial screening for CD in children with T1DM. However, the proportion of patients found to be negative is very small. We included DQ2.2/- as a positive result, given the evidence from typing studies in proven patients with CD, but clarification and proper definition of what is the relative risk of specific haplotypes and haplotype combinations would be extremely helpful for clinicians and patients. Although if negative, these patients can be excluded from future CD testing, the cost-effectiveness still depends on the cost of genotyping and the frequency/duration of subsequent CD serology screening. Finally, our findings suggest that rationalising the duration/frequency of serum tTG screening to twice in the first 5 years after a diagnosis of T1DM may prove an efficient screening strategy.
